# Sitting *vs.* squatting: a scoping review of toilet postures and associated health outcomes

**DOI:** 10.1186/s12889-025-23379-8

**Published:** 2025-07-02

**Authors:** Niloofar Rahgoshay, Mohammad Rahdar, Laleh Nikoo, Hadi Daneshmandi

**Affiliations:** 1 Student Research Committee, Shiraz University of Medical Sciences, Shiraz, Iran; 2 Department of Ergonomics, Shiraz University of Medical Sciences, Shiraz, Iran; 3 Research Center for Health Sciences, Institute of Health, Shiraz University of Medical Sciences, Shiraz, P.O. Box: 71645-111, Iran

**Keywords:** Design, Ergonomic, Health outcome, Scoping review, Sitting, Squatting, Toilet

## Abstract

**Background/objective:**

The comparison between squatting and sitting toilets involves complex health, ergonomic, and design considerations. This scoping review aims to synthesize evidence on the digestive, musculoskeletal, and ergonomic health outcomes associated with squatting and sitting toilet postures, while also exploring related health impacts (e.g., urinary and cardiovascular) and design considerations.

**Methods:**

This scoping review was conducted on January 1, 2024, searching Scopus, PubMed, and Web of Science, following Preferred Reporting Items for Systematic Reviews and Meta-Analyses extension for Scoping Reviews (PRISMA-ScR) guidelines. Inclusion criteria were defined using the patient/population, intervention, comparison and outcomes (PICO) framework. Two reviewers independently screened titles, abstracts, and full texts, supplemented by hand searches in Google Scholar and Science Direct, and snowballing. Methodological quality was appraised using Joanna Briggs Institute (JBI) checklists.

**Results:**

Forty-two studies were analyzed. Squatting may reduce digestive strain and enhance bowel evacuation, potentially benefiting constipation, while sitting toilets may increase bowel-related issues but provide comfort for specific populations, such as older adults. Musculoskeletal outcomes vary, with squatting linked to strain in vulnerable groups and sitting toilets reducing joint stress when ergonomically designed. Ergonomic innovations, such as adjustable seats and non-slip surfaces, show promise in improving comfort and minimizing health risks. Methodological limitations, including small sample sizes and inadequate control of confounders, preclude definitive conclusions.

**Conclusion:**

Squatting and sitting toilet postures differentially influence digestive health, musculoskeletal strain, and sanitation, shaped by individual needs, cultural practices, and environmental factors. Practical implications include developing adjustable, hygiene-focused, culturally sensitive toilet designs to enhance public health. Longitudinal studies with robust methodologies are needed to clarify chronic health impacts and optimize user-centered toilet design solutions.

**Supplementary Information:**

The online version contains supplementary material available at 10.1186/s12889-025-23379-8.

## Introduction

Toilet posture during defecation significantly impacts public health, yet it remains underexplored in research and policy. Variations in toilet habits, particularly the shift from squatting to sitting postures, have been associated with digestive, musculoskeletal, and ergonomic health outcomes [1–3]. Squatting is defined as a posture where the hips are lowered close to the ground, with knees fully flexed and feet flat, typically associated with traditional pit or squat toilets. Sitting is defined as a posture on a raised toilet seat, with hips and knees at approximately 90-degree angles, as seen in Western-style flush toilet [4]. From an evolutionary standpoint, squatting aligns more closely with the natural human defecation position, potentially benefiting digestive health [2]. Studies indicate that when in a squatting position, the anorectal angle increases to about 100–110 degrees, causing the rectum to straighten. This anatomical change facilitates the process of defecation, making it easier [4]. Moreover, squatting toilets have been reported to potentially reduce the risk of colon and prostate diseases [5, 6] and digestive disorders such as constipation and hemorrhoids [7]. Simultaneously, researchers have noted that squatting can result in lower limb injuries. Specifically, studies indicate that the sustained pressure on the knees and leg muscles while squatting can lead to fatigue and discomfort in the back, legs, and even throughout the entire body [8–10], potentially compromising stability and balance [11]. The ergonomic characteristics of squatting toilets are primarily determined by foot size and the positioning associated with the squatting posture [12]. In their research, Tucker et al. investigated the squatting position and demonstrated that the maximum distance achieved while squatting can disrupt the body’s balance [13]. This suggests that stability and comfort are crucial factors for squatting toilets [14]. Defecating in a sitting position is generally easier than squatting; however, it often demands more time and energy. The absence of pressure between the body and thighs while seated may contribute to rectal muscles adhering to the rectum, potentially leading to blockage. Some evidence suggests that Western populations have experienced a rise in bowel-related issues, including constipation, hemorrhoids, and irritable bowel syndrome [15]. The ergonomic design of seating toilets is determined by the dimensions suitable for sitting, with the anorectal angle typically ranging from 80 to 90 degrees in this position [16]. When sitting, leaning forward with proper leg support may lead to a passive rise in intra-abdominal pressure while potentially relaxing the pelvic floor. It has been proposed that toilet aids could serve as effective tools to help individuals achieve a squatting position [15]. The choice of toilet posture may influence the prevalence of certain diseases, particularly among aging populations where sitting toilet use is increasing [17]. Some research suggests that squatting may be more effective for defecation [18]. However, cultural and religious preferences, such as those in some Asian and Middle Eastern communities, may also influence the adoption of squatting toilets, alongside personal comfort and habit [19]. Nevertheless, some clinicians recommend that older adults use sitting toilets for safety, suggesting both squatting and sitting toilets be available at home to accommodate diverse user needs [4]. A large-scale survey indicated that, while most respondents reported using sitting toilets at home, many women expressed a preference for squatting toilets [20]. Similarly, a field survey in Taiwan found that 86% of participants believed squatting toilets may better address their health needs compared to other toilet types [14].

The increasing prevalence of health issues related to squatting and sitting toilets can be linked to suboptimal ergonomic design. This gap may stem from limited integration of medical and physiological knowledge into toilet design. The significance of body posture during urination and defecation, along with its impact on health, presents a complex challenge for researchers and designers [18].

Despite growing evidence, the relationship between toilet posture and health remains underexplored. This scoping review aims to synthesize evidence on the digestive, musculoskeletal, and ergonomic health outcomes associated with squatting and sitting toilet postures, while also exploring related health impacts (e.g., urinary and cardiovascular) and design considerations.

## Methods

The study was approved by the ethics committee of Shiraz University of Medical Sciences (Ethics Code: IR.SUMS.SCHEANUT.REC.1402.156).

### Search strategy

This scoping review follows a systematic approach aligned with the Preferred Reporting Items for Systematic Reviews and Meta-Analyses extension for Scoping Reviews (PRISMA-ScR) guidelines [21]. The PRISMA-ScR checklist is included in Supplementary 1. Inclusion criteria were defined using the patient/population, intervention, comparison and outcomes (PICO) framework: Population (P) included individuals of any age, gender, or health status using squatting or sitting toilets; Intervention (I) involved squatting or sitting defecation postures; Comparison (C) included studies comparing these postures or no comparison; Outcomes (O) encompassed health and ergonomic impacts; and Study design (S) included all original studies, such as cross-sectional, case-control, quasi-experimental, or qualitative. Eligible studies were peer-reviewed and English-language with no restrictions. Exclusion criteria comprised non-peer-reviewed publications (e.g., editorials, conference abstracts), non-English articles, and studies unrelated to toilet posture or health outcomes. Two independent reviewers conducted a systematic search of the existing literature on January 1, 2024 in Scopus, PubMed, and Web of Science [22]. Various combinations of terms were utilized, taking advantage of the advanced features of each database, along with Medical Subject Headings (MeSH) and verified online dictionaries such as Cambridge, Collins, and Merriam-Webster, to encompass the entire scope of the study, including synonyms and different applications of scientific terminology. The main search structure consisted of three groups of keywords as follows:

- G1: (Toilet OR lavatory OR lavatories OR defecation OR latrine OR excretion OR evacuation)
- G2: (bowel OR colon OR sitting OR squat OR pain OR discomfort OR Cancer)
- G3: (Convenience OR comfort OR seat OR safety OR sanitation OR sanitary).

Complete search strings are provided in Supplementary 2. Throughout all stages of the review, disagreements and doubts between the reviewers were discussed to reach a consensual agreement. When consensus could not be reached, expert advice was sought.

### Screening

Data screening was conducted by two reviewers using EndNote Version 8. In the screening phase, duplicate entries were first eliminated, and the remaining studies were organized according to their document types. Two reviewers subsequently assessed the titles and abstracts of these studies. A total of 76 articles were selected for full-text access. However, three studies were excluded at this point because the files were unavailable or the PDFs could not be obtained. Multiple attempts were made to reach out to the authors via email in an effort to acquire these studies.

### Hand search

To guarantee that no pertinent articles were missed, we included a hand search phase in our methodology. Two independent reviewers conducted further searches in additional databases, including Google Scholar and Science Direct. We also implemented a snowballing strategy, which involved reviewing all references cited in the studies that were included. This approach led to the addition of eight more studies to our collection.

### Data extraction

During the data extraction phase, various relevant elements from each article were taken into account. The gathered information encompassed the author/s, year, country, study design, type of toilet, populations, number of samples, objectives, methods and main outcomes. Two authors independently extracted data from the selected studies.

### Critical appraisal

The Joanna Briggs Institute (JBI) checklist critical appraisal checklists were employed to assess the methodological quality of the included studies. Both qualitative and quantitative research underwent a comprehensive assessment process, tailored to their respective study designs, as outlined by JBI. For critical appraisal purposes, responses to the checklist questions were categorized as “yes,” “no,” “unclear,” or “not applicable” [23]. Since the overall methodological quality of the studies did not influence their inclusion in the scoping review, we decided against developing a weighting scheme to calculate a total quality score. This decision aligns with the scoping review methodology, which prioritizes mapping all available evidence regardless of quality to provide a comprehensive overview, as recommended by JBI and PRISMA-ScR guidelines [21, 24]. Instead, we focused on evaluating quality based on individual criteria. The assessment was conducted independently by two authors.

## Results

Following the full-text review, 34 articles were identified as eligible for inclusion in the study. A subsequent hand search, including snowballing, added eight more studies, resulting in a total of 42 articles incorporated into the data extraction phase.

### Description of included studies

**Fig. 1:**
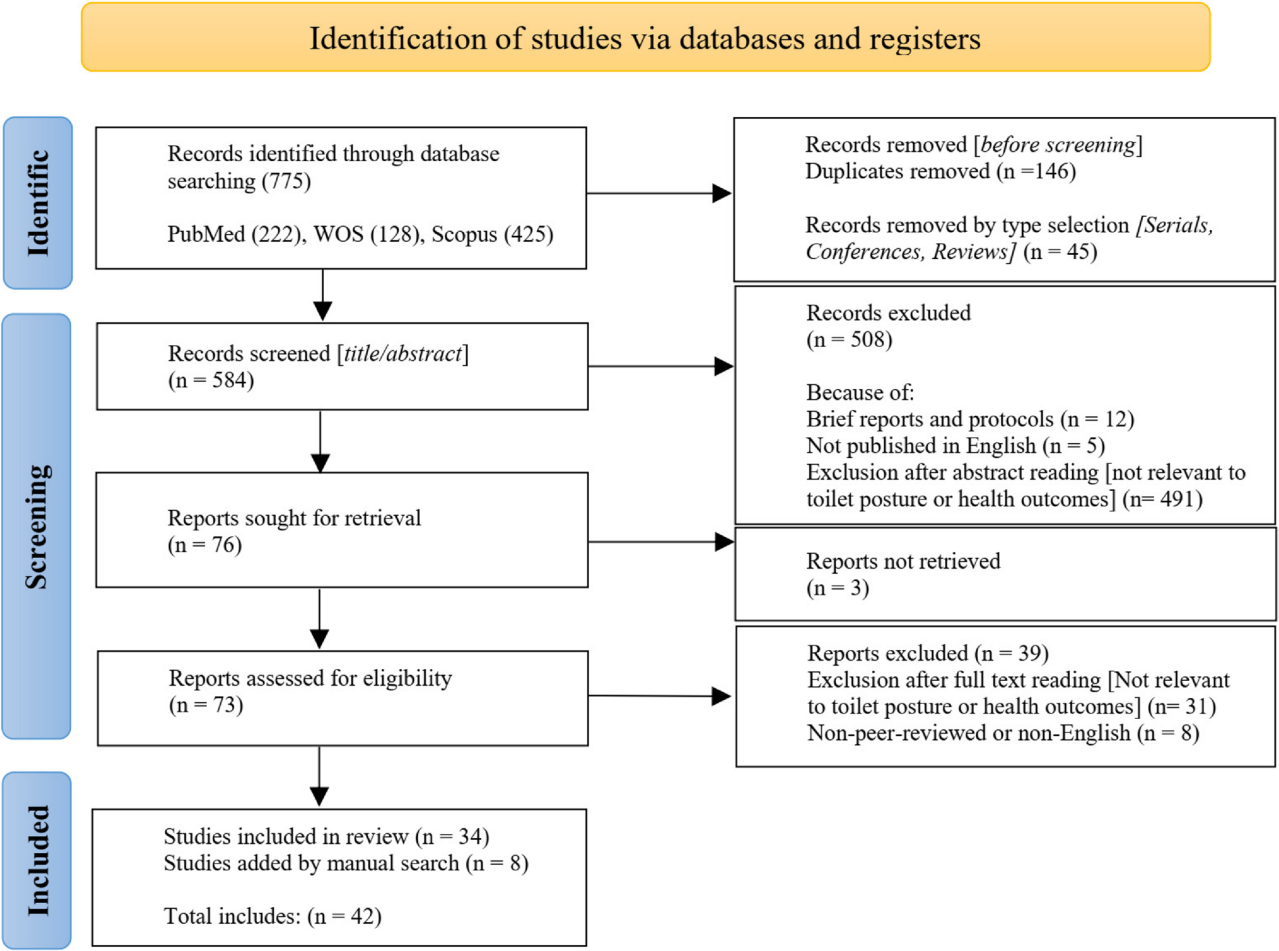
Flow chart diagram of the review

**Fig. 2:**
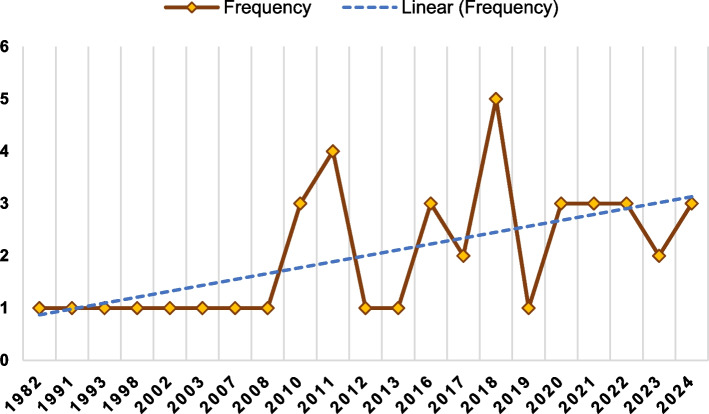
A timeline of the publication years

**Fig. 3:**
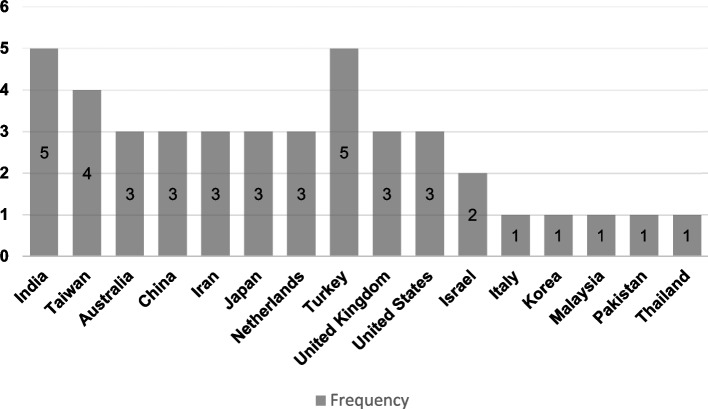
Geographic distribution of the studies

A total of 42 studies were analyzed, focusing on various aspects of sitting and squatting toilets. The PRISMA-ScR flow diagram illustrates the outcomes at each stage of the selection process (Fig. 1). Among these studies, 18 studies concentrated on sitting toilets, while 8 studies examined squatting toilets; additionally, 16 studies analyzed both types. The publication years of these studies ranged from 1982 in the United Kingdom [25] to 2024 in Taiwan and Turkey [26–28]. A timeline of the publication years is presented in Fig. 2. Sample sizes varied significantly, from a minimum of 3 participants in Israel [29] to a maximum of 2721 in Turkey [28]. The geographic distribution of the studies is shown in Fig. 3.

### Results of the included studies

The scoping review analyzed 42 studies categorized into six groups based on their health impacts, including digestive, pelvic floor, urinary, environmental, cardiovascular, and musculoskeletal health. Another subgroup also considered for practical toilet designs. An overview of the included studies is provided in Appendix Table 1.

#### Digestive health

Eleven studies examined various aspects of defecation posture and its implications for gastrointestinal health, focusing on conditions such as functional constipation, hemorrhoids, anal fissures, and colonic diverticulosis (CD). Gupta et al. investigated the association between defecation posture and diet concerning functional constipation among Indian children. They found a significant link between sitting toilet use and mixed diets with functional constipation [30]. Takano et al. introduced “The Thinker” position (bending the upper body forward and placing the elbow on the knee) as an alternative defecation method for patients struggling with evacuation issues, reporting improved efficiency compared to traditional sitting; however, further investigation is required to confirm its effectiveness [31]. Sakakibara et al. compared abdominal pressure and anorectal angles across three positions—sitting, hip-flex sitting, and squatting—finding that squatting resulted in a straighter recto-anal canal requiring less strain for defecation [32]. Similarly, Sikirov’s research found lower straining effort while squatting compared to sitting positions [2]. Giuliani et al. identified a linear association between prolonged toilet sitting time and severity of hemorrhoids [33]. Uzun et al.’s study revealed no significant correlation between preferred toilet type and hemorrhoids among Turkish hospital patients [34]. Ahmed et al.’s evaluation of defecation postures in chronic anal fissure treatment indicated more favorable outcomes with modified commode squatting patterns compared to standard sitting or squatting [35]. Shekokar et al. found that patients using sitting postures reported more relief from rectal bleeding than those using squatting positions [36]. Ozturk et al.’s examination of colonic diverticulosis prevalence indicated higher sitting toilet use among affected individuals [17]. Buldukoglu et al. reported that sitting toilet use was a risk factor for colonic diverticulosis, with an odds ratio of 3.36 (95% CI: 1.684–6.705, *P* = 0.001) in multivariate logistic regression analysis [27]. Sohrabi et al.’s assessment found no significant association between sitting posture during defecation and colorectal cancer risk [6].

#### Pelvic floor health

Two studies examined the relationship between toileting behaviors and pelvic floor health. Kose et al. investigated the impact of toileting behaviors on the natural course of anterior vaginal wall prolapse (AVWP) in 75 women who underwent surgery for symptomatic AVWP. Their findings reported that squatting was associated with increased severity of AVWP symptoms, linked to higher intra-abdominal pressure, and a shorter time from symptom onset to surgery compared to sitting [37]. Lam et al. evaluated pelvic floor positioning during defecation straining in 52 patients across three positions: left lateral, sitting, and squatting. The results showed that there was no significant difference in pelvic floor descent between sitting and squatting positions during straining, and squatting did not reduce the risk of pelvic nerve damage associated with chronic straining [38].

#### Urinary health

Four studies explored the relationship between toilet types and their impact on urinary tract infections (UTIs) and urinary excretion efficiency. Parasuraman et al. assessed the correlation between defecation posture and UTI risk in a representative adolescent sample in Malaysia. The results showed that most participants preferred squatting toilets in public to avoid contact with toilet bowls and 10.7% reported a history of UTIs [39]. Yang et al. investigated the effects of sitting and non-sitting postures on uroflowmetric parameters in women, finding no significant differences in post-void residual (PVR) urine or uroflowmetric outcomes among postures; however, there was a longer delay time to void in the semi-squatting position compared to sitting and crouching. The study reported that 88.9% preferred non-sitting postures in public settings [40]. Additionally, Rane et al. evaluated squat and lean-forward postures on micturition, reporting no significant differences in uroflowmetric parameters between postures, with squatting ability varying among individuals [41]. Moore et al.’s study found that crouching was associated with reduced urinary flow and increased residual urine volume [42].

#### Environmental health

Three studies examined the health implications of different toilet types and environmental health. Mahdavinejad et al. investigated the impact of toilet type on infection transmission by comparing sitting and squat toilets. Their examination of 40 toilets for pathogenic microorganisms found that sitting toilets had lower levels of germs, although no significant differences were found in gastrointestinal and non-gastrointestinal pathogens [43]. Ali et al. investigated bioaerosol emissions from squat and bidet toilets following flushing. Higher concentrations of Staphylococcus aureus bioaerosols were measured in bathrooms equipped with squat toilets, particularly during defecation postures. The study measured lower bioaerosol concentrations under turned-on air-exhaust ventilation compared to when ventilation was turned off. In hand washing posture, lower exposure levels were observed, which coincided with a greater distance from the toilet. Additionally, the measured disease burden decreased with increased time intervals after flushing, across both toilet types and exposure scenarios [44]. In the COVID-19 pandemic, Pan et al. assessed public perceptions of toilet cleanliness and preferences regarding squatting versus seated toilets. Their survey showed that 91% preferred squatting toilets, while 72% expressed concerns about contamination in public facilities. The study reported that many respondents experienced water splashes during flushing and suggested improvements in hygiene, such as foot-controlled devices to cover toilets during flushing [45].

#### Cardiovascular health

Chakrabarti et al. explored the correlation between squatting posture during defecation and stroke incidence in India. They found that 36% of strokes occurred while individuals were in a squatting position, with over half of hemorrhagic strokes occurring in this posture. The study reported significant increases in blood pressure associated with squatting in both healthy individuals and those with hypertension [46].

#### Musculoskeletal health

Two studies examined musculoskeletal injuries associated with squat toilets, specifically focusing on injuries to the Achilles tendon caused by the edge of the squat toilet seat. Mohsin et al. investigated patients who sustained foot injuries due to squatting toilet seats. All patients received comprehensive wound irrigation and debridement, followed by the repair of the cut Achilles tendon, other tendons, and neurovascular structures [47]. Dar et al. examined the mechanism of tendoachilles laceration resulting from squat toilet use, suggesting that adopting sitting toilets may help avoid these injuries [48].

### Solutions

Design studies consisted of three main groups: 1. Biomechanical aspects, 2. Assistive devices and 3. Toilet design. Detailed findings were further indicated.

#### Biomechanical aspects

Five studies examined biomechanical considerations in toilet design. Lee et al. aimed to quantify the kinetic characteristics of transfers to and from a sitting toilet using bilateral swing-away grab bars. The study found that using bilateral swing-away grab bars resulted in decreased peak moments at the leg joints during both the transition from standing to sitting and from sitting to standing on the toilet. The study reported minimal differences in peak joint moments when comparing grab bar widths ranging from 0.330 m to 0.381 m and heights from 0.787 m to 0.838 m [49]. Lee et al. examined how changes in toilet seat angles and heights affected movements and ground reaction forces during transitions from sitting to standing. The study reported that adjusting toilet height and angle was associated with increased stability [50]. Snijders et al. assessed the suitability of higher sitting toilets for elderly individuals, finding that an increase in height above the standard was associated with a greater hip angle and reduced postural mobility [51]. Musch explored the concept of lower point-supported toilets, reporting that in this lower sitting position, the center of gravity shifts closer to the feet, resulting in reduced force required to stand up, particularly for smaller individuals whose feet can touch the ground [52]. Lustig et al. investigated biomechanical and microcirculatory responses of buttock tissues during prolonged sitting on toilet seats. Their findings showed that extended sitting was associated with an increased risk for pressure injuries (PrIs), influenced by seat design. Specialized toilet seat cushions reduced tissue stress by up to 88% [29].

#### Assistive devices

Five studies examined various aspects of defecation postures and the use of assistive devices, such as footstools and defecation postural modification devices (DPMDs), in relation to bowel health. Trieu et al. assessed the impact of footstools on defecatory posture in patients with constipation, finding that footstool use altered the angle between the spine and femur but did not result in improvements in subjective or objective measures of simulated defecation [53]. Modi et al. found that DPMDs were associated with increased bowel emptiness and reduced straining, with healthy participants reporting shorter bowel movement durations in a crossover study [54]. Edgar et al.’s study reported that using a footstool reduced defecation time, averaging 55.5 seconds compared to 113.4 seconds in the seated position. Healthy participants reported a strain rating of 1.4 with the footstool versus 2.5 for the seated position [55]. Takano et al. found that footstools were associated with facilitated defecation when the upper body was bent forward, reducing evacuation time and increasing rectal pressure in older patients [56]. Dekker et al.’s research explored support types in toilet environments for elderly and disabled individuals, finding that preferences varied based on individual needs rather than body dimensions; vertical supports were preferred for both standing and sitting [57].

#### Toilet design

Nine studies examined the design, usability, and health aspects of various toilet types. Yu reported that the importance of integrating assessments based on “convenience,” “comfort,” “reliability,” “value,” “safety,” and “efficiency” to create toilets that align with human physiological and psychological needs [4].

##### Squatting

Lee’s study assessed differences in comfortable squatting postures between men and women, finding that men had a comfortable outward foot angle of 40.56° compared to 28.99° for women [26]. Chen et al.’s study examined optimal design parameters for squat toilets among Taiwanese and Southeast Asian women, finding no significant difference in squatting stability between the two groups and identifying a preferred span between feet (SBF) of 16 cm [58]. Cai et al.’s case study on public squatting-type toilets found that nearly half of participants preferred squatting over sitting and identified a 15° slope as preferred for squatting comfort [14].

##### Sitting

McClelland et al.’s research reported a standard seat height of 0.4 m as preferred based on user preferences [25]. Tharbthong et al. reported that a toilet seat height of 110.0% of an individual’s lower leg length (LLL) was suitable for older adults, as it was associated with optimal rectus femoris muscle activity, reduced time taken to rise, and higher user satisfaction compared to heights of 100.0% and 120.0% [59]. Yokoyama et al.’s study utilized Kansei Engineering to evaluate toilet seat prototypes based on user comfort; findings showed that wider seats were associated with increased satisfaction, leading to the development of a new model named “TRES” with a wider back and middle seat area, a forward-tilted surface to assist elderly users in standing up, and a water-efficient design that eliminates the traditional tank [60]. Mehdi’s research identified a mismatch between modern toilet seat designs and human anthropometry and proposed a conceptual framework for redesigning toilet seats [61]. Başıbüyük et al. found that toilet seat designs generally do not accommodate the anthropometric measurements of older adults, based on data from 2721 individuals aged 65 years and older in Turkey [28].

### Critical appraisal of the included studies

**Fig. 4:**
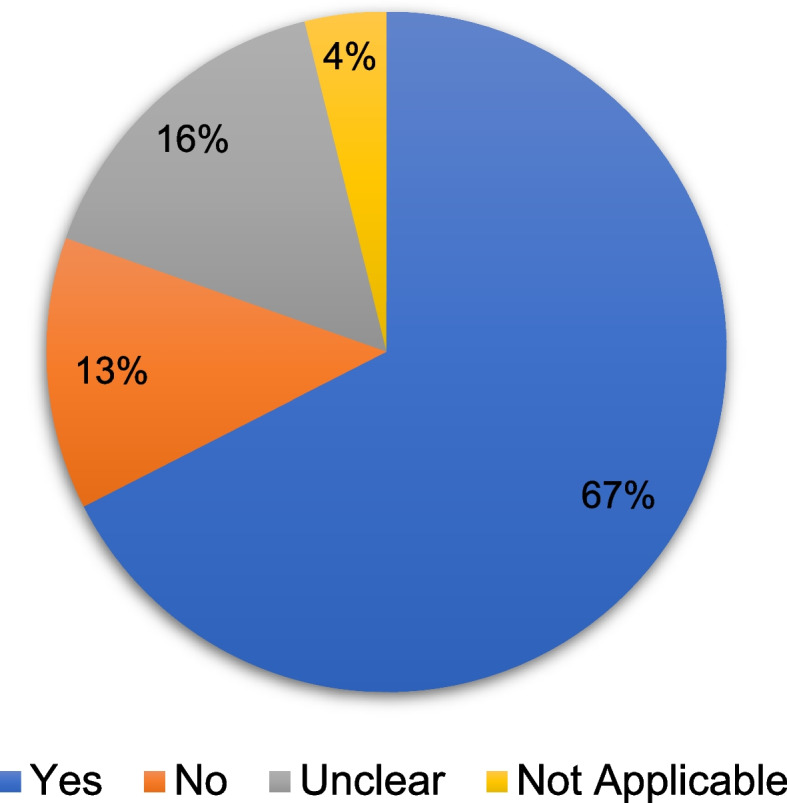
The quality of articles based on the JBI critical appraisal

The critical appraisal of eligible studies, outlined in Appendix Table 2. Approximately 67.49% of responses to the JBI checklist criteria were affirmative, while 12.94% were negative, 15.70% unclear, and 3.87% not applicable. A visual summary of the critical appraisal results is provided in Fig. 4. Cross-sectional studies (20 studies) showed strengths in appropriate statistical analysis (Q8: 90% Yes) and detailed description of study subjects (Q2: 80% Yes), but only 45% identified confounding factors (Q5), and 40% adequately managed them (Q6). Quasi-experimental studies (13 studies) demonstrated consistent outcome measurement (Q6: 100% Yes) and clear cause-and-effect relationships (Q1: 100% Yes) but mostly lacked control groups (Q2: 23.07% Yes), limiting their ability to establish causality. Case-control studies were robust in group comparability (Q1: 100% Yes) and statistical analysis (Q10: 100% Yes) but failed to manage confounders (Q7: 0% Yes). The single RCT achieved 69.23% affirmative responses but had limitations in blinding (Q4/Q5: No). The cohort study achieved 72.72% affirmative responses, with strengths in managing confounding factors (Q4/Q5: Yes) but weaknesses in ensuring participants were free of outcomes at the start (Q6: No). Case series studies provided clear reporting of clinical information (Q7: 100% Yes) but had deficiencies in reporting clinic/site information and statistical analysis (Q9/Q10: 0% Yes). The qualitative study scored 60% affirmative responses, while expert opinion studies achieved 75% affirmative responses but varied in literature congruence (Q6: 50% Yes).

## Discussion

### Description of studies

This scoping review aims to synthesize evidence on the digestive, musculoskeletal, and ergonomic health outcomes associated with squatting and sitting toilet postures, while also exploring related health impacts (e.g., urinary and cardiovascular) and design considerations. The sample sizes of included studies varied widely, from as few as 3 participants in Israel to a maximum of 2721 in Turkey, indicating diverse research methodologies and participant engagement across different regions. The analysis indicates that there is more research activity in the Middle East, particularly in countries like India and Turkey. This difference can be attributed to cultural practices surrounding toilet use, health concerns related to bowel function, and varying focuses on design innovations across regions.

### Discussion of the included studies

**Fig. 5:**
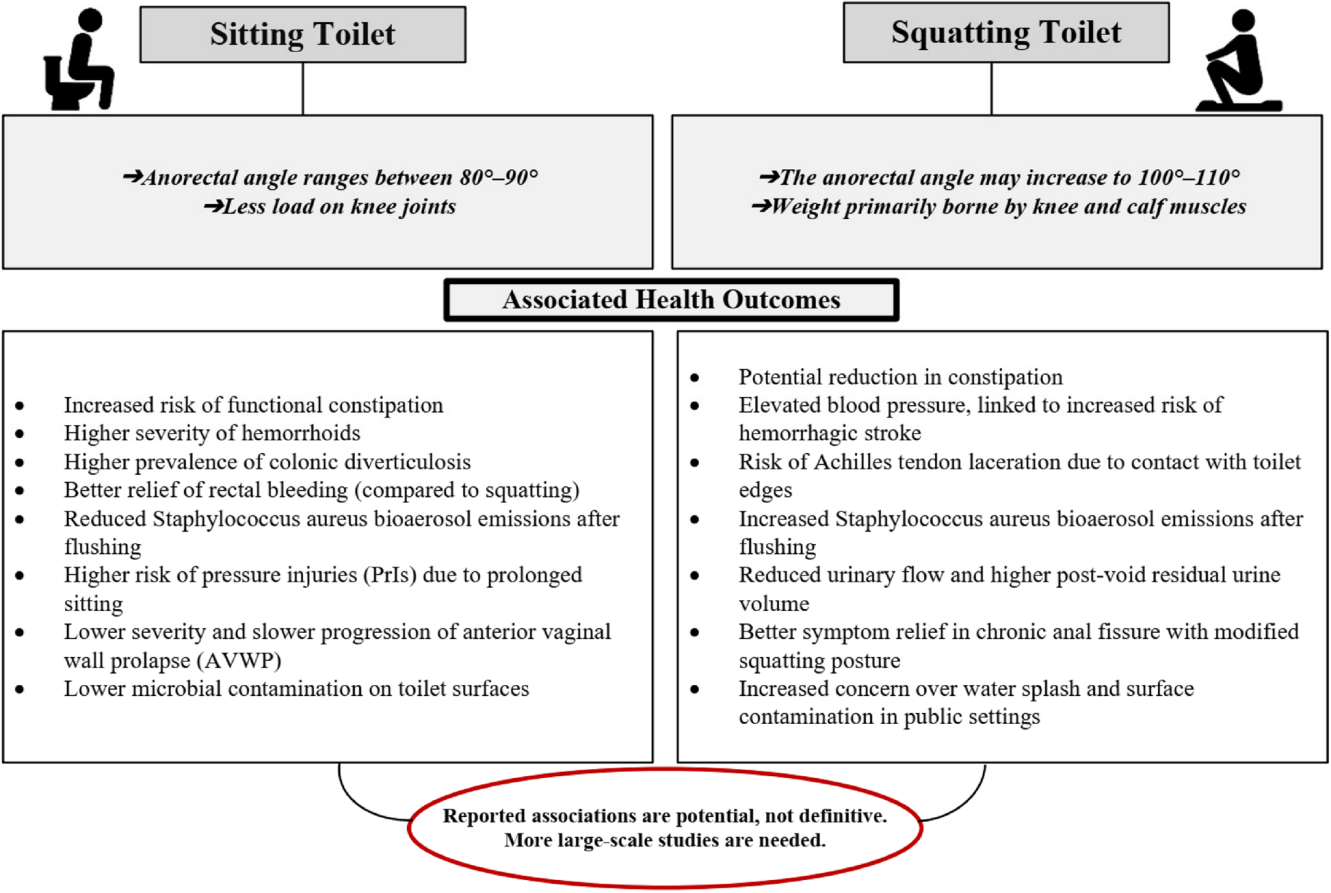
A conceptual framework illustrating the proposed causal pathways between toilet posture and health outcomes

The scoping review analyzed 42 studies categorized into six groups based on their health impacts, including digestive, pelvic floor, urinary, environmental, cardiovascular, and musculoskeletal health. Another subgroup also considered for practical toilet designs. Figure 5 showed a conceptual framework illustrating the proposed causal pathways between toilet posture and health outcomes.

#### Digestive health

The relationship between defecation posture and gastrointestinal health varies across studies. According to a conceptual framework, biomechanical (e.g., anorectal angle), physiological (e.g., straining effort), and behavioral (e.g., duration of toilet use) factors mediate this relationship. Some studies report that squatting is associated with potential benefits for digestive health compared to sitting toilets [4, 8–10]. For example, studies found reduced straining with squatting, requiring less abdominal pressure and effort, which was associated with alleviated strain on the rectal muscles and improved bowel evacuation. Studies also reported that squatting was linked to improved bowel movement efficiency, potentially associated with benefits for individuals with constipation or hemorrhoids [30–32, 62]. A review by Bhattacharya et al. reported that squatting was associated with increased engagement of abdominal muscles, linked to complete bowel evacuation [18]. A review by Suri et al. reported that the sitting position was associated with increased straining and incomplete bowel evacuation [63]. However, the relationship between toilet type and gastrointestinal disorders is complex; while posture plays a role, other factors such as diet, lifestyle, and individual anatomy must also be considered [30]. Shekokar et al. reported that sitting toilets were associated with a lower risk of rectal bleeding in specific contexts [36]. Ozturk et al. [17] and Buldukoglu et al. [27] reported that sitting toilet use was associated with an increased risk of colonic diverticulosis, suggesting that sitting postures may contribute to elevated luminal pressure in the colon. Findings vary across studies; two studies report no significant correlation between toilet type and certain health conditions. Uzun et al. found no link with hemorrhoids, but its focus on patients with gastroenterological complaints and cross-sectional design suggest a need for cohort studies [34]. Sohrabi et al. found no association with colorectal cancer, though retrospective design and non-population-based controls weaken the evidence [6]. This variation in findings indicates a need for larger-scale research to clarify the relationship between bowel health and posture modification. Many studies have methodological limitations, including small sample sizes, lack of longitudinal data, and weaknesses in managing confounding factors such as diet or physical activity. Factors such as body type, muscle strength, and individual physiology may influence the effectiveness of different toilet postures.

#### Pelvic floor health

The relationship between toileting behaviors and pelvic floor health varies across studies. According to a conceptual framework, defecation posture influences pelvic floor outcomes through biomechanical factors (e.g., intra-abdominal pressure) and physiological factors (e.g., pelvic nerve strain). Two studies examined this relationship, reporting differing outcomes for specific pelvic floor conditions [37, 38]. Kose et al. reported that squatting was associated with increased severity of AVWP symptoms, linked to higher intra-abdominal pressure, and a shorter time from symptom onset to surgery compared to sitting [37]. Lam et al. reported that squatting did not reduce pelvic floor descent during defecation straining compared to sitting and was not associated with a reduced risk of pelvic nerve damage from chronic straining [38]. These findings are based on only two studies with methodological limitations, including small sample sizes (75 and 52 participants, respectively) and context-specific designs. Individual factors, such as specific medical conditions, may influence the choice of toileting position.

#### Urinary health

The relationship between toileting postures and urinary health outcomes, such as urinary tract infections and urinary excretion efficiency, remains inconclusive due to inconsistent findings across studies. According to a conceptual framework, biomechanical factors (e.g., pelvic positioning) and behavioral factors (e.g., hygiene-driven posture preferences) may influence urinary health, though empirical evidence is limited by methodological constraints. Parasuraman et al. reported that 10.7% of participants had a history of UTIs and suggested that squatting postures, preferred in public settings to avoid toilet bowl contact, may be associated with reduced UTI risk due to minimized surface contact [39]. However, this correlation is not definitive due to methodological limitations, such as the study’s reliance on self-reported data and failure to control for confounders. Similarly, Yang et al. found that 88.9% of women preferred non-sitting postures in public toilets, likely driven by hygiene concerns, but observed no significant differences in PVR urine across sitting, semi-squatting, and crouching postures. However, the study noted small sample size (*N*=45) and use of a modified toilet for safety limit the generalizability of these findings, underscoring the need for larger, more robust studies [40]. Rane et al. observed that squatting ability varied among individuals but reported no significant differences in uroflowmetric parameters between postures [41]. In contrast, Moore et al. found that crouching was associated with reduced urinary flow and increased residual urine volume, potentially increasing UTI susceptibility [42]. Collectively, these studies highlight that while non-sitting postures are often preferred for hygiene reasons, their impact on urinary health is not well-established. The inconsistent findings and methodological flaws—such as small sample sizes, reliance on self-reports, and weak study designs—limit the ability to draw definitive conclusions. The preference for squatting in public settings suggests a need for improved toilet design and public health education to address hygiene concerns.

#### Environmental health

The relationship between toilet types and infection transmission remains complex, with studies suggesting potential differences but lacking conclusive evidence due to methodological limitations. Mahdavinejad et al. found that sitting toilets had lower levels of pathogenic microorganisms compared to squat toilets, but the failure to control for confounders weakens the claim that sitting toilets inherently reduce infection risks [64]. In line with this, Ali et al. reported that squat toilets released higher concentrations of Staphylococcus aureus bioaerosols after flushing, particularly under conditions with turned-off air-exhaust ventilation, thereby contributing to increased infection risk. However, the study’s specific focus on S. aureus and limited ventilation scenarios may constrain the generalizability of its findings to broader settings [44]. Pan et al. assessed public perceptions during the COVID-19 pandemic and noted that while a majority preferred squatting toilets, many expressed concerns about contamination in public settings. The study’s small and non-randomized sample restrict the generalizability of these findings [45]. These findings suggest that while sitting toilets may offer advantages in reducing microbial presence under certain conditions, squat toilets remain prevalent due to cultural preferences, particularly in developing regions. World Health Organisation (WHO) highlights the need for improved sanitation facilities globally, as a significant portion of the population still relies on inadequate systems, including squat toilets without proper hygiene measures. This lack of sanitation contributes to health risks, including the transmission of diseases through fecal contamination. Squat toilets, commonly used in many developing regions, often lack features such as lids and U-traps, which can lead to increased exposure to pathogens through aerosolized droplets during flushing. The WHO has recommended that users take precautions in these settings to minimize health risks [65].

#### Cardiovascular health

Chakrabarti et al. observed that 36% of strokes, including over half of hemorrhagic strokes, occurred in a squatting position, primarily during defecation, suggesting a potential link with elevated blood pressure in specific contexts, such as India, where squat toilets are prevalent [46]. These findings indicate that squatting may contribute to transient blood pressure spikes, potentially acting as a trigger for stroke in vulnerable individuals, such as those with hypertension. However, the evidence is insufficient to broadly implicate squatting as a direct cause of stroke. Given the cultural preference for squat toilets in many regions, including India, public health strategies should focus on identifying at-risk populations and promoting safer practices, such as monitoring blood pressure in hypertensives who use squat toilets.

#### Musculoskeletal health

The association between squat toilets and musculoskeletal injuries, particularly Achilles tendon lacerations, underscores the need for safer toilet designs, though evidence is limited by small-scale studies. Mohsin et al. [47] and Dar et al. [48] reported that foot slips in squat toilets can cause severe injuries, including complete or partial Achilles tendon tears, with some cases requiring complex surgical interventions such as microvascular repair or plastic surgery. However, these findings are based on small sample sizes (26 and 12 patients, respectively), with Mohsin et al.’s single-center study [47] limiting generalizability. The lack of detailed methodological controls in Dar et al. [48] further restricts the ability to draw broad conclusions. These studies suggest that the level design of squat toilets, which allows the foot to slip and contact the toilet rim, contributes to these injuries. Cultural preferences for squat toilets in regions like India necessitate practical solutions that balance safety and tradition. Public health efforts could focus on modifying squat toilet designs, such as adding non-slip surfaces or raised edges, to minimize injury risks.

### Solutions

#### Biomechanical aspects

According to a conceptual framework, toilet posture influences biomechanical health through force distribution, postural stability, and tissue loading. Studies suggest potential benefits of specific toilet designs for user well-being [29, 49–52]:

- Bilateral swing-away grab bars may reduce joint moments during toilet transfers, as shown by Lee et al. [49].
- Adjusting toilet seat height and angle may reduce forward-backward sway, potentially improving stability, as suggested by Lee et al. [50].
- Toilet designs with hand grips for disabled individuals may facilitate defecation and standing, as explored by Snijders et al. [51].
- Lower point-supported toilets, proposed by Musch, may reduce the force needed to stand by shifting the center of gravity closer to the feet [52].
- Specialized toilet seat cushions, as demonstrated by Lustig et al., reduce tissue stress by up to 88%, potentially preventing pressure injuries [29].
- Holistic care approaches incorporating pressure injury prevention devices across care environments are supported by Lustig et al.’s findings [29].

These findings are limited by methodological constraints, including small sample sizes, focus on healthy or young cohorts, and exploratory designs, which restrict generalizability. Toilet designers may prioritize adjustable seats and grab bars to improve biomechanical outcomes, pending further research.

#### Assistive devices

According to a conceptual framework, toilet posture influences bowel health through biomechanical factors, including anorectal angle, rectal pressure, and postural stability. Assistive devices, such as footstools and DPMDs, aim to optimize these factors to improve defecation outcomes, though their effectiveness varies across populations. Studies collectively indicate potential benefits but are limited by methodological constraints [53–57]. For instance, Trieu et al. observed that footstools altered the spine-femur angle in constipated patients without improving simulated defecation measures [53]. In contrast, Modi et al. found that DPMDs enhanced bowel emptiness and reduced straining in healthy volunteers, with shorter bowel movement durations [54]. Similarly, Edgar et al. reported that footstools decreased defecation time and effort in healthy individuals [55]. Takano et al. noted that footstools reduced evacuation time and increased rectal pressure in older patients with obstructed defecation, particularly when the upper body was bent forward [56]. Dekker et al. highlighted preferences for vertical supports among elderly and disabled individuals to aid sitting and standing [57]. However, these studies face limitations, including small sample sizes (ranging from 14 to 53 participants), non-randomized designs, reliance on subjective scales, and cohorts often limited to healthy or elderly populations, which restrict generalizability to broader clinical contexts Physical therapists and healthcare professionals may consider footstools as part of tailored interventions to reduce defecation time and effort, pending further validation. Toilet manufacturers could explore integrating adjustable vertical supports into designs to meet diverse user needs. Elderly care facilities and public restrooms may benefit from customizable support options to enhance accessibility. Krishnan’s review underscores that musculoskeletal challenges in elderly individuals often hinder squatting, with current squat-assist platforms inadequately addressing lower limb difficulties. A cost-effective, mechatronic assistive system could improve support for optimal defecation postures, though such innovations require further development and rigorous testing [66].

#### Toilet design

Several studies highlight the importance of ergonomic principles in toilet design, suggesting that both squatting and sitting toilets require tailored features to accommodate different users’ needs [14, 25, 60]. Lee found that men and women have distinct comfortable squatting postures, suggesting specific shapes for toilet designs to enhance stability and comfort [26]. To address the ergonomic and anthropometric needs of older adults, Başıbüyük et al. conducted a comprehensive anthropometric study involving 2721 individuals aged 65 and over in Turkey. The study provided data on key body dimensions—including the sitting hip breadth (403 mm), buttock-popliteal length (512 mm), and popliteal height (405 mm)—to inform the ergonomic design of toilet facilities. Based on their findings, the authors recommend that toilet seat height correspond to the 5 th percentile female popliteal height (405 mm) to promote safety and ease of use, thereby reducing the risk of falls. Additionally, they advise that toilet seat width should accommodate the 95 th percentile female sitting hip breadth (403 mm) to ensure adequate support and postural stability for older users [28]. Tharbthong et al. conducted a study to determine the optimal toilet seat height for older Thai adults, utilizing anthropometric data from 342 individuals aged 60 and above. In the experimental phase, they evaluated three seat height levels equivalent to 100.0%, 110.0%, and 120.0% of each participant’s LLL. The results indicated that a seat height corresponding to 110.0% of LLL—equivalent to 40.92 cm for females and 43.27 cm for males—was the most suitable, aligning with the 95 th percentile of the population’s LLL. This height was associated with the highest user satisfaction, reduced quadriceps (rectus femoris) muscle effort compared to 100.0% LLL, and facilitated a smoother and quicker sit-to-stand transition. The range of 100.0–120.0% LLL was deemed ergonomically optimal, as it minimized hip and knee joint extension, thereby improving balance and potentially reducing the risk of falls in older adults [59]. Other Studies like those by McClelland et al. [25] and Mehdi [61] indicate that modern toilet designs may fail to meet ergonomic standards, potentially leading to hygiene issues and user discomfort, but Mehdi’s conceptual framework lacks empirical validation. While some studies suggest a trend towards modern sitting toilets, Cai et al.’s study found that 86% of participants in Taipei preferred squatting due to better sanitation outcomes [14]. These findings suggest a need for toilet designs that balance ergonomic and cultural considerations to promote health and prevent disease.

### Critical appraisal of the included studies

The critical appraisal, as detailed in Section “ Critical appraisal of the included studies”, indicates moderate methodological rigor, with 67.49% affirmative responses, but highlights notable gaps that affect the reliability of findings. Key limitations include inadequate control of confounding factors, limited use of control groups, and restricted sample sizes. These issues compromise the internal validity of several studies, potentially leading to biased conclusions about the causal relationship between defecation posture and health outcomes. Consequently, observed associations may stem from methodological flaws rather than genuine effects, warranting cautious interpretation.

### Potential bias

**Fig. 6:**
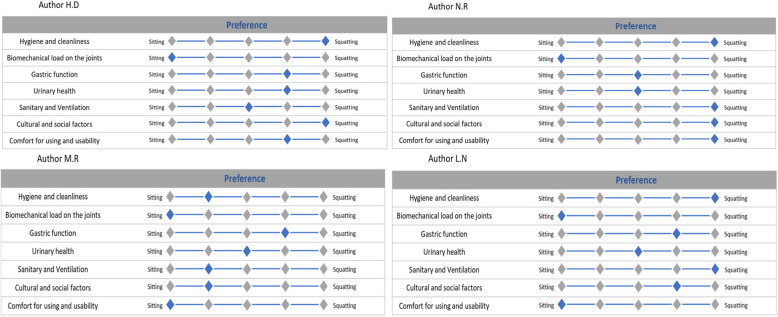
Authors’ perspectives on different aspects of each toilet type

To address methodological concerns and reduce potential biases in our own analysis and interpretation, we completed the following scale to help minimize confirmation bias and to gain insights into each authors’ perspectives on different aspects of each toilet type (Fig. 6).

### Strengths and limitations

This review presents several strengths, primarily its comprehensive and systematic approach, guided by the PRISMA-ScR framework. It encompasses a wide range of health outcomes and includes studies with diverse methodologies, enriching the overall interpretation of the findings. However, several limitations should be acknowledged. There is a risk of publication bias and diversity in the quality of the reviewed studies. Additionally, the shortage of long-term studies exploring the chronic health effects of various toilet types and postures represents a significant gap in our understanding. Conflicting evidence regarding the effectiveness of different defecation postures further highlights the lack of consensus in the literature, emphasizing the need for more research in this domain. Lastly, the restriction to English-language articles may have excluded relevant studies published in other languages, particularly from regions where squatting toilets are culturally prevalent, such as Asia. This language restriction could introduce bias by omitting evidence from non-English-speaking populations, potentially limiting the global applicability of the findings.

### Practical recommendations for designers, public health officials, and policymakers

To translate the findings of this review into actionable outcomes, designers, public health officials, and policymakers should adopt evidence-based strategies to enhance toilet functionality and public health. Designers should integrate ergonomic principles, such as adjustable seat heights and hybrid squatting-sitting models, to minimize strain on digestive and musculoskeletal systems while aligning with anatomical needs. Public health officials should promote hygiene-focused features, including enhanced ventilation and touchless systems, to reduce infection risks associated with public restrooms. Policymakers should advocate for inclusive toilet facilities that accommodate cultural preferences, such as squatting options in regions where they are favored, and ensure accessibility through assistive technologies for individuals with mobility challenges. These measures can optimize sanitation practices, improve user comfort and health, and align facilities with diverse societal needs.

### Suggestions for future research

To optimize toilet design for health and comfort, future research should investigate ergonomic principles, such as the impact of toilet height and posture on musculoskeletal strain and digestive outcomes, across diverse populations, including various age groups and mobility levels. Additionally, studies should explore the development of affordable assistive technologies to enhance accessibility and efficiency for individuals with mobility challenges. To ensure robust findings, large-scale, multi-center studies involving diverse populations are needed to confirm results and enhance generalizability. Studies should also control for confounders, such as diet and hygiene practices, to strengthen causal inferences. Longitudinal studies should examine the chronic health effects of different toilet types and defecation postures. Lastly, research should investigate how cultural beliefs and attitudes shape toilet use preferences and health outcomes, informing inclusive design solutions that align with diverse societal needs.

## Conclusion

This scoping review reveals that squatting and sitting toilet postures differentially impact digestive health, musculoskeletal strain, and sanitation, with findings shaped by individual anatomical needs, cultural practices, and environmental contexts. Squatting may enhance digestive outcomes by reducing straining and improving bowel evacuation, while sitting toilets, when ergonomically designed, can minimize musculoskeletal stress and enhance stability, particularly for older adults or those with mobility challenges. Cultural preferences, such as the prevalence of squatting in regions like Iran and India for hygiene and tradition, underscore the importance of context in toilet use. However, inconsistent findings, driven by methodological limitations and short-term study designs, highlight the need for longitudinal research to clarify chronic health impacts. Ergonomic innovations, including adjustable seat heights, non-slip surfaces, and assistive devices like footstools, show promise in mitigating health risks and improving user comfort. These insights advance knowledge by emphasizing the need for tailored toilet designs that integrate anatomical, cultural, and hygiene considerations to optimize public health outcomes, while calling for robust, long-term studies to guide evidence-based design solutions.

## Supplementary Information

Supplementary Material 1.

## Data Availability

The datasets used and/or analysed during the current study are available from the corresponding author on reasonable request.

## Appendix

**Table 1:** Overview of the included studies

Author/Year	Country/Toilet type	Article type	Population/Sample	Aim	Method	Main outcome
Lee 2024 [26]	Taiwan/squatting	Cross-sectional	Healthy participants/60 (30 Females)	To design a squatting toilet that conformed to the natural squatting posture of different sexes	The study measured the oscillation stability index (OSI), postures, and comfort levels for 24 different toilet designs to provide a holistic view of their usability.	New design solutions proposed for men and women to support better stability.
Başıbüyük et al. 2024 [28]	Turkey/Sitting	cross-sectional	Elderly people/2721 (1530 Females)	To evaluate the ergonomic design of toilets and bathroom equipment for older adults using anthropometric measurements	Fourteen anthropometric measurements were assessed, with body dimensions described by min, max, mean, SD, and 5 th, 25 th, 50 th, 75 th, 95 th percentiles.	Advises designers and manufacturers to adapt bathroom products to older adults’ body characteristics to enhance person-environment fit.
Buldukoglu et al. 2024 [27]	Turkey/Sitting and squatting	Cross-sectional	Patients undergoing colonoscopy/929 (465 Females)	To investigate whether toilet type (sitting vs. squatting) influences diverticulosis development, hypothesizing that squatting toilets reduce the risk by lowering colonic pressure.	A one-page questionnaire on demographics, bowel habits, and diverticulosis factors was used, with colonoscopy results matched to responses.	The study confirmed that sitting toilets are a risk factor for diverticulosis, supporting the hypothesis and laying the groundwork for future research.
Trieu et al. 2023 [53]	Australia/Sitting	Randomized Trial	Patients with constipation/41 (38 Females)	To assess the alterations in defecatory posture, and the changes in simulated defecation with the use of a footstool	Three rectal balloon expulsion tests in randomized order through these stages: 1) no footstool, 2) 7-inch, and 3) 9-inch footstools were conducted for each participant.	Using footstool led to changes in defecatory posture, but it did not improve subjective or objective measures of simulated defecation.
Gupta et al. 2023 [30]	India/Sitting and squatting	Case-Control	Children 2-18 years old/300	To investigate whether defecation posture and diet are associated with functional constipation in Indian children	150 children with functional constipation compared to a similar control group in terms of defecation posture and toilet type.	It was noted that defecation in sitting posture and mixed diets is associated with functional constipation.
Uzun et al. 2022 [34]	Turkey/Sitting and squatting	Cross-sectional	Patients suffer from Hemorrhoid/142 (81 Females)	To investigate the relationship between the development of hemorrhoid disease and toilet habits	Diagnostic tests for hemorrhoid via colonoscopy and Presence of constipation was evaluated according to Rome IV criteria and the patients were asked to fill out a short questionnaire after the procedure.	There was no relationship between the preferred toilet type and hemorrhoid disease.
Kose et al. 2022 [37]	Turkey/Sitting and squatting	Cohort	Women who underwent surgery for symptomatic AVWP/75	To investigate the effect of toileting behaviors on the natural course of anterior vaginal wall prolapse (AVWP)	Participants divided into two groups according to their defecation position: Sitting or squatting. A set of related questionnaires used to assess their symptoms and feelings.	Squatting may increase the severity of symptoms related to prolapse more than sitting.
Ali et al. 2022 [44]	China/Sitting and squatting	Cross-sectional	**_**	To examine and compare the field measurements of Staphylococcus aureus bioaerosol emission characteristics in squat and bidet toilets	Infection emission characteristics were measured in different heights and two ventilation scenarios. Then a quantitative health risk model was performed for different exposed populations concerning the annual probability of infection and disease burden.	The highest bioaerosol concentration was in bathroom with squat toilet. Moreover, the infection risk was higher in adult males.
Pan et al. 2021 [45]	China/Squatting	Cross-sectional	Volunteers/134 (96 Females)	To determine whether the cleanliness of public toilets was a concern to society during Coronavirus disease 2019 (COVID-19) pandemic, and if squatting toilet was preferred to a seated design	A questionnaire was designed and posted on WeChat contact groups of the investigators.	A simple foot-controlled device could be installed to cover public squatting toilets and reduce COVID-19 contamination.
Mehdi 2021 [61]	Iran/Sitting and squatting	Conceptual design	**–**	To investigate the mismatch distance between the inner surface of a toilet seat and the human body	Modeling process developed in SolidWorks which incorporated various geometrical features and anthropometric data from an analysis of toilet seat designs.	Some adjustments in the size and shape of the toilet seat form hygienic point of view were adjusted.
Chen et al. 2021 [58]	Taiwan and southeast Asia/Squatting	Cross-sectional	Women/140	To determine optimal design parameters for squat toilets by collecting SBF data for participants squatting in their most subjectively comfortable posture	Span between feet (SBF) data of 100 females in their comfortable squatting posture has provided. Then 40 participants underwent a stability test under two SBF conditions to compare squatting stability levels.	There was no difference in stability between the two SBFs. The comfortable SBF can be considered as an optimal toilet width parameter because of its constant stability.
Mohsin et al. 2020 [47]	India/Squatting	Case series	Acute injuries around ankle due to slipping of one of their feet in squatting toilet/26 (7 Females)	To report on the severity of open Achilles tendon injury caused by squatting toilet and to evaluate the outcome of surgical treatment of these injuries	Wound irrigation and debridement followed by repair of cut tend Achilles, other tendons, and neurovascular structures was performed for the injured cases. All the complications and secondary procedures required were recorded.	Foot injuries involving tendons and neurovascular structures caused by squatting type toilet may require microvascular tissue transfer for definitive wound management.
Giuliani et al. 2020 [33]	Italy/Sitting	Cross-sectional	Non-obese patients with internal or external hemorrhoids/52 (19 Females)	To assess how common is the habit to spend a long time on the toilet in patients seen for hemorrhoidal disease	A short questionnaire divided into four classes was filled based on patients’ level of hemorrhoid case. Mean time spent on the toilet for each group was calculated and then assessed in relation to their disease severity.	Time spent on the toilet and degree of hemorrhoids seemed to be linked by linear association.
Tharbthong et al. 2020 [59]	Thailand/Sitting	Cross-Sectional	Elderly people/342 (190 Females)	To determine the main factors affecting older people as they rise from a toilet seat and to identify the suitable toilet seat height for this population.	Anthropometric data from older adults were used to design a mock-up toilet with seat width based on hip breadth and seat heights at 100.0%, 110.0%, and 120.0% of lower leg length, testing muscle activity, rising time, thigh pressure, and satisfaction.	The optimal toilet seat height for older adults, for ergonomic purposes, was 110.0% of lower leg length) LLL(, equivalent to the 95 th percentile of male and female LLL.
Modi et al. 2019 [54]	United States/Sitting	Quasi-Experimental	Healthy volunteers/52 (21 Females)	To evaluate the influence of defecation postural modification devices (DPMDs) on normal bowel patterns	Prospective crossover study performed including real-time collection of data regarding bowel movements (BM) for 2 weeks without DPMDs and 2 weeks with them.	DPMDs positively influenced BM duration, straining pattern, and complete evacuation of bowels according to this study.
Lee et al. 2018 [49]	United States/Sitting	Quasi-Experimental	Healthcare professionals/11 (9 Females)	To evaluate the impact of bilateral swing-away grab bars, height, and width variety on peak joint moments at the ankle, knee, and hip during stand-to-sit and sit-to-stand transfers.	Healthy subjects performed stand-to-sit and sit-to-stand transfers with and without grab bars, testing nine configurations that varied in height and width.	Ranges of grab bar configurations reduce moment demands on the leg joints and thus decrease difficulty and required lower limb muscle effort to perform the transfers.
Yu 2018 [4]	China/Sitting and squatting	Conceptual design	**_**	To analyze the structured problems in the existing toilets that cause irrational phenomena in postures and methods of use based on universal design rules.	Comparing two common types of toilets based on their Ergonomic features and physiological effects on users.	The new toilet design should be based on Ergonomics and convenience across other physiological and universal aspects.
Takano et al. 2018 [56]	Japan/Sitting	Quasi-Experimental	Patients experiencing constipation/53 (25 Females)	To determine the efficacy of adding a footstool to help facilitate defecation in patients with fecal outlet obstruction	Cinedefecography was performed with and without a foot stool. Anorectal angle, perineal plane distance, and puborectalis length during rest and straining in both positions were measured from the radiographs.	Using footstool is more efficient method for defecation. However, the upper body bent forward position is also important.
Ozturk et al. 2018 [17]	Turkey/Sitting	Cross-sectional	Patients who underwent colonoscopy/757 (340 Females)	To determine the prevalence, location and clinical features of colonic diverticulosis (CD) and its relation with posture	Patients followed a uniform diet and bowel preparation regimen before their colonoscopy. Medical histories, toilet usage, postures and toilet use duration were recorded.	Sitting during defecation seems to increase the risk of colonic diverticulosis.
Lustig et al. 2018 [29]	Israel/Sitting	Quasi-Experimental	3 (2 Females)	To investigate the immediate biomechanical and microcirculatory responses of the buttock tissues to toilet sitting pressure injury (PrI) and deep tissue injuries (DTIs)	Creating a model to analyze sitting effects on bare and cushioned toilet seats, six model variants were developed based on MRI data from a spinal cord injury subject. Then analyzed tissue stresses using numerical simulations.	Specialized toilet seat cushions can reduce this risk of pressure injury, by lowering tissue exposures and internal stresses by 88% and maintaining reduced interface pressures.
Shekokar et al. 2017 [36]	India/Sitting and squatting	Quasi-Experimental	Patients with anorectal bleeding/60	To compare the role of sitting and squatting posture in anorectal bleeding during defecation	The main treatment involved bathing with application of an ointment around the anus twice a day for 15 days for treatment group. The progress was tracked weekly for a month with additional follow ups.	Patient suffered with anorectal bleeding should use sitting toilet for defecation instead of squatting toilet.
Edgar et al. 2017 [55]	United Kingdom/Sitting	Quasi-Experimental	Staff and university students/33 (9 Females)	To investigate the effectiveness of defecation postures and anal cleaning method on movement and sanitation	Volunteers recorded the time taken to empty their bowels in two positions: seated on a pedestal toilet and seated with feet raised on a footstool to mimic squatting.	Using a footstool reduced the defecation time from 113.4 seconds to 55.5 seconds. Participants rated the effort required as 2.5 (moderate) when seated and 1.4 (easy) when using a footstool.
Takano et al. 2016 [31]	United States/Sitting	Quasi-Experimental	Patients with constipation/22 (17 Females)	To assess the influence of “The Thinker” position on defecation	Patients who received 200 ml of liquid barium and 50 ml barium paste as enema were asked to sit on a commode, and lateral pelvic films were taken during the pushing phase. Design variables were measured during straining in both “the thinker” and “the sitting” positions.	“The Thinker” position seems to be a more efficient method for defecation than the sitting position especially in the cases of constipation.
Parasuraman et al. 2016 [39]	Malaysia/Sitting and squatting	Cross-sectional	Adults/551 (334 Females)	To assess the correlation between defecation posture and risk of urinary tract infection (UTI) rates	The customized online questionnaires were used to assess Knowledge and attitude toward the relation of defecation posture and UTI.	Squatting method of defecation prevents gastrointestinal disorders and improves the muscular strength in lower limbs.
Lee et al. 2016 [50]	Korea/Sitting	Quasi-Experimental	Healthy adults/20 (14 Females)	To examine the effects of changes in the angle and height of the toilet seat on movements and ground reaction forces in the feet	Tumble Forms Wedges were placed on the toilet seat to create different angles and heights. Each subject repeated the standing motion three times for each angle, with two-minute breaks in between.	Toilet seat angle and height increase affected forward/backward swaying during standing up, but did not affect the ground reaction force and side-to-side swaying.
Ahmed et al. 2013 [35]	Pakistan/Sitting and Squatting	Quasi-Experimental	Patients with chronic anal fissure/100 (43 Females)	To evaluate the effect of defecation postures on the outcome of chronic anal fissure	Patients were assessed before and after changing their defecation postures and compared sitting and squatting postures versus commode squatting pattern.	The modified commode squatting posture has the highest success rate for the treatment of chronic anal fissure.
Sohrabi et al. 2012 [6]	Iran/Sitting and Squatting	Case-control study	Patients with colorectal adenocarcinoma (CRC)/200 (73 Females)	To examine the association between using squatting versus sitting toilets and CRC risk	Participants completed a questionnaire regarding their toilet usage patterns over ten-year periods, along with socioeconomic factors, smoking history, aspirin consumption and constipation history.	The study did not support an appreciable role for using sitting toilets as risk factors for CRC.
Snijders et al. 2011 [51]	Netherlands/Sitting	Cross-sectional	Elderly people/14	To question the suitability of a higher toilet for the elderly	Biomechanical measures such as pelvic floor geometry was done using in-vivo and in-vitro measurements. Analysis of anthropometric data was implemented for the optimal range of adjustable toilet height.	Proper height and Foot contact is essential for standing up after toileting which indicates the need for toilet seat at popliteal height. Hand grips in front of the impaired should be a basic convenience.
Musch et al. 2011 [52]	Netherlands/Sitting and squatting	Cross-sectional	20	To develop a more comfortable squatting posture and improve hygiene and adaptation for elderly users	A foam mock-up of a lower point-supported toilet is created to simulate the squatting posture. A group of 20 participants evaluates the mock-up by providing feedback.	The design reduced the arm force required for the activity. For squatting postures, lower point supported toilets could offer improved comfort, hygiene, and safety.
Mahdavinejad et al. 2011 [43]	Iran/Sitting and squatting	Cross-sectional	40 toilets (20 sitting type and 20 squatting type)	To obtain the role of toilet type in transmission of infections	Frequency of pathogenic germs and their types were determined and compared between two groups of toilets with one-year manufacturing date.	Using of sitting toilets should be encouraged to reduce the rate of bio-transmission and prevention of gastrointestinal, respiratory, and genital infection.
Dar et al. 2011 [48]	India/Squatting	Case series	Patients with cut wounds over the Achilles tendon/12 (3 Females)	To report injuries of the Achilles tendon caused by a unique mechanism caused by the edge of toiled seat	Patients received empirical antibiotic therapy followed by targeted antibiotics based on sensitivity reports. They were immobilized and then gradually rehabilitated.	Copious washes and broad-spectrum antibiotics help control the infection in such potentially contaminated wounds. Such injuries could be avoided using a sitting toilet system.
Yokoyama et al. 2010 [60]	Japan/Sitting	Qualitative	**_**	To explain the developmental process of the new Kansei toilet design, namely TRES	Japanese Kansei method was implemented to collect data about the comfort feeling of the subjects who used 8 toilets from different companies. Physical dimensions and body pressure on the new toilet seat were measured.	All the subjects were satisfied while when sitting on the new design (TRES)
Yang et al. 2010 [40]	Taiwan/Sitting, semi-squatting, crouching	Cross-sectional	Healthy female students/45	To compare the effects of non-sitting and sitting postures for micturition on urinary flow and post-void residual (PVR) urine and investigating their preference	Related variables of uroflowmetry were measured and the Data were collected in three different postures in sitting, semi-squatting, and crouching forms. After each voiding PVR was estimated by researcher.	Voiding in the three postures did not differ in terms of PVR and five of six uroflowmetric parameters.
Sakakibara et al. 2010 [32]	Japan/Sitting	Quasi-Experimental	Healthy volunteers/6 (5 Females)	To compare three positions for defecation be measuring abdominal pressure and the anorectal angle simultaneously using anorectal video manometry	Measurements were obtained by urodynamic computer and 9 F and 8 F catheters for abdominal pressure and rectal pressure. After filling the subjects’ anorectum with contrast medium, they were asked to defecate. The process repeated for 3 times with 3 postures.	Results showed that the greater the hip flexion achieved by squatting, the straighter the recto-anal canal will be, and accordingly, less strain will be required for defecation.
Rane et al. 2008 [41]	Australia/Squatting	Quasi-Experimental	Volunteers/125 Females	To evaluate the effects of posture on micturition in the lean forward and squatting positions	Subjects randomly allocated sequences of two acts of micturition in anteverted and the squatting position. Bladder scanning was done before and after micturition.	Near squat position marginally improved flow rates over the anteversion position.
Dekker et al. 2007 [57]	Netherlands/Sitting	Quasi-Experimental	Elderly people/14 (9 Females)	To gain insight into preferred types of supports, associated range of heights and distances for toilet use	Subjects were asked to sit down and get up using supports at various positions. They utilized the supports for their toilet use and report their comfort to the researcher.	No significant relationship was found between the use of supports and gender differences, stature, body mass and grip strength. All the participants favored using vertical supports for both rising and sitting down.
Sikirov 2003 [2]	Israel/sitting and squatting	Quasi-Experimental	Volunteers/28 (14 Females)	To compare the straining forces applied when using toilet in sitting or squatting position during defecation	Subjects instructed to record time spent in six consecutive bowel movements in each position.	The relative time and the sensation of satisfactory bowel emptying in sitting defecation posture necessitates excessive expulsive effort compared to the squatting posture.
Chakrabarti et al. 2002 [46]	India/Squatting	Cross-sectional	Hypertensive patients/100 (31 Females)	To assess the validity of observations about the increase of strokes while squatting in Indian population	For Subjects with hypertension, arm BP was measured after 5 min rest in supine position on bed and thereafter on squatting on the floor for at least 1 min.	Squatting and specially defecation in squatting posture would tend to cause a heightened adrenergic response which may be implicated in stroke onset in subjects at risk.
Cai et al. 1998 [14]	Taiwan/Squatting	Cross-sectional	Students/80 (9 Females)	To demonstrate the feasibility of an ergonomic approach to product development and design using a new toilet design	Then the anthropometric investigation of the subjects was conducted. Finally, the design process of a new toilet developed with different footstep slopes.	Results showed that the ergonomic approach is feasible recommended to be adopted in the process of design in such facilities. 15-degree slope was found less fatiguing while used for squatting position.
Lam et al. 1993 [38]	Australia/Sitting and squatting	Cross-sectional	Patient of anorectal physiology lab/52	To measure the position of the pelvic floor during defecation straining in the sitting and squatting position	The position of the perineum was measured with respect to the plane of the ischial tuberosities. Recording was taken at rest and during straining in sitting and squatting positions.	Squatting during defecation straining does not cause a reduction in pelvic floor descent compared with normal condition. It was shown that squatting during defecation would not help in preventing pelvic nerve damage.
Moore et al. 1991 [42]	United Kingdom/Sitting	Cross-sectional	Gynecology patients/528 Women	To determine the relationship between voiding posture and urinary dysfunctions and uroflowmetric factors	Participants surveyed about crouching on toilet and then undergone uroflowmetry in both crouching and sitting positions.	Average urine flow rate reduced and residual urine volume increased during crouching position. Knowing about individual postures is essential for abnormality diagnosis.
McClelland et al. 1982 [25]	United Kingdom/Sitting	Quasi-Experimental	Volunteers/205 (127 Females)	To examine appropriate position and configuration for toilet seat	Continuous steps for both seat height and angle with 5 types of seats was done.	It is recommended that the height of a toilet seat should be 40 cm. Seat angle is not a critical factor in this subject.

**Table 2:** Critical appraisal of the included studies

Type	Study	Y=Yes, N=No, U=Unclear, N/A=Not Applicable							Q_1_	Q_2_		Q_3_			Q_4_	Q_5_	Q_6_	Q_7_	Q_8_
Cross sectional	Lee 2024 [26]								Y	Y		Y			Y	U	U	Y	Y
	Başıbüyük 2024 [28]								Y	Y		Y			Y	U	N	Y	Y
	Buldukoglu 2024 [27]								Y	Y		Y			Y	Y	Y	Y	Y
	Uzun 2022 [34]	Q1: Inclusion criteria clearly defined? Q2: Study subjects described in detail? Q3: Valid and reliable exposure measurement? Q4: Objective, standard criteria for measuring the condition? Q5: Confounding factors identified? Q6: Dealing with confounding factors? Q7: Outcomes measured in valid and reliable way? Q8: Appropriate statistical analysis?							Y	Y		U			Y	Y	Y	Y	Y
	Ali 2022 [44]								Y	Y		Y			Y	Y	Y	Y	Y
	Pan 2021 [45]								U	Y		U			N	N	N	U	Y
	Chen 2021 [58]								Y	Y		Y			Y	U	U	Y	Y
	Giuliani 2020 [33]								Y	Y		U			Y	U	N	U	Y
	Tharbthong 2020 [59]								Y	Y		Y			Y	U	N	Y	Y
	Ozturk 2018 [17]								Y	Y		Y			Y	Y	Y	Y	Y
	Parasuraman 2016 [39]								U	Y		U			U	N	N	N	Y
	Lee 2016 [50]								Y	Y		Y			Y	U	N	Y	Y
	Snijders 2011 [51]								Y	Y		Y			Y	U	N	U	U
	Musch 2011 [52]								N	N		U			U	Y	U	U	U
	Mahdavinejad 2011 [43]								Y	N/A		Y			Y	N	N	Y	Y
	Yang 2010 [40]								Y	Y		Y			Y	Y	Y	Y	Y
	Chakrabarti 2002 [46]								Y	Y		U			Y	Y	Y	U	Y
	Cai 1998 [14]								U	N		Y			Y	U	U	Y	Y
	Lam 1993 [38]								N	U		U			N	Y	Y	U	Y
	Moore 1991 [42]								N	Y		Y			U	Y	Y	U	Y
Type	Study	Y=Yes, N=No, U=Unclear, N/A=Not Applicable					Q_1_		Q_2_	Q_3_		Q_4_			Q_5_	Q_6_	Q_7_	Q_8_	Q_9_
Quasi Experimental	Modi 2019 [54]						Y		N	Y		Y			Y	Y	Y	N/A	Y
	Takano 2018 [56]	Q1: Clear “Cause” and “Effect”? Q2: Any control group? Q3: Similar Comparison groups? Q4: Similar treatment/care among groups? Q5: Multiple measurement of the outcome? Q6: Same way to measure outcome in groups? Q7: Reliable measuring? Q8: Differences in follow-up described and analyzed? Q9: Appropriate statistical analyze?					Y		N	U		U			Y	Y	Y	N/A	Y
	Lustig 2018 [29]						Y		N	N		N			Y	Y	Y	N/A	Y
	Lee 2018 [49]						Y		N	Y		Y			Y	Y	Y	N/A	Y
	Shekokar 2017 [36]						Y		Y	Y		Y			Y	Y	Y	Y	Y
	Edgar 2017 [55]						Y		N	N		Y			N	Y	U	N/A	Y
	Takano 2016 [31]						Y		N	U		Y			Y	Y	Y	N/A	Y
	Ahmed 2013 [35]						Y		Y	U		Y			N	Y	U	U	U
	Sakakibara 2010 [32]						Y		N	Y		Y			Y	Y	Y	N/A	Y
	Rane 2008 [41]						Y		N	Y		U			Y	Y	Y	N/A	Y
	Dekker 2007 [57]						Y		N	Y		Y			N	Y	Y	N/A	Y
	Sikirov 2003 [2]						Y		Y	Y		Y			Y	Y	Y	N/A	Y
	McClelland [25] 1982						Y		N	Y		Y			N	Y	Y	N/A	Y
Type	Study	Y=Yes, N=No, U=Unclear, N/A=Not Applicable				Q_1_	Q_2_		Q_3_	Q_4_		Q_5_			Q_6_	Q_7_	Q_8_	Q_9_	Q_10_
Case Control	Gupta 2023 [30]					Y	Y		Y	U		Y			U	N	Y	Y	Y
	Sohrabi 2012 [6]					Y	Y		Y	U		Y			Y	N	Y	Y	Y
		Q1: Comparable groups? – Q2: matched groups? – Q3: Same criteria in groups? – Q4: Standard and reliable measuring? Q5: Same measurement in groups? – Q6: Confounding factors identified? – Q7: Dealing with confounding factors? – Q8: Standard and reliable assessment for outcomes? – Q9: long exposure period? – Q10: Appropriate statistical analysis?																	
Type	Study		Q_1_	Q_2_	Q_3_	Q_4_	Q_5_		Q_6_	Q_7_		Q_8_			Q_9_	Q_10_	Q_11_	Q_12_	Q_13_
RCT	Trieu 2023 [53]		Y	U	Y	N	N		Y	U		Y			Y	Y	Y	Y	Y
		Q1: True randomization? – Q2: Concealed allocation? – Q3: Treatment similar to baseline? – Q4: Blind assignment? – Q5: Blind delivering of treatment? – Q6: Identical treatment instead of intervention? – Q7: Blind outcome assessment? – Q8: Same outcome measuring in groups? – Q9: Reliable measurement? – Q10: Differences in follow-up described and analyzed? – Q11: Analyzed in groups? – Q12: Appropriate statistical analysis? – Q13: Appropriate trial design?																	
Type	Study	Y=Yes, N=No, U=Unclear, N/A=Not Applicable			Q_1_	Q_2_	Q_3_	Q_4_			Q_5_		Q_6_		Q_7_	Q_8_	Q_9_	Q_10_	Q_11_
Cohort	Kose 2022 [37]				Y	Y	Y	Y			Y		N		Y	Y	N/A	U	Y
		Q1: Similar groups? – Q2: Assigned both exposed and unexposed? – Q3: Valid and reliable exposure measurement? – Q4: Confounding factors identified? Q5: Dealing with confounding factors? – Q6: Participants free of outcome at the start? – Q7: Valid and reliable outcome measurement? – Q8: Long follow-up? – Q9: Lost to follow-up described and explored? – Q10: Strategies to address incomplete follow-up? – Q11: Appropriate statistical analysis?																	
Type	Study	Y=Yes, N=No, U=Unclear, N/A=Not Applicable				Q_1_	Q_2_	Q_3_			Q_4_	Q_5_			Q_6_	Q_7_	Q_8_	Q_9_	Q_10_
Case Series	Mohsin 2020 [47]					Y	Y	Y			Y	N			Y	Y	Y	U	U
	Dar 2011 [48]					Y	Y	N			Y	Y			N	Y	Y	U	N/A
		Q1: Clear inclusion criteria? – Q2: Standard, Reliable way to measure conditions? – Q3: Valid methods for identification? – Q4: Consecutive inclusion of participants? Q5: Complete inclusion of participants? – Q6: Clear reporting of demographic information? – Q7: Clear reporting of clinical information? – Q8: Clear reporting of the follow-up outcomes? – Q9: Clear reporting of presenting clinic/site information? – Q10: Appropriate statistical analysis?																	
Type	Study	Y=Yes, N=No, U=Unclear, N/A=Not Applicable				Q_1_	Q_2_	Q_3_		Q_4_		Q_5_		Q_6_		Q_7_	Q_8_	Q_9_	Q_10_
Qualitative	Yokoyama 2010 [60]					Y	Y	Y		N		Y		Y		N	N	Y	U
		Q1: Congruity of perspective and methodology? – Q2: Congruity of methodology and objectives? – Q3: Congruity of methodology and data collection? – Q4: congruity of methodology and representation and analysis? Q5: Congruity of methodology and interpretation of results? – Q6: Cultural of theoretical bias? – Q7: Influence of researcher on research? – Q8: Representation of participants’ voices? – Q9: Ethical approval? – Q10: Conclusion report flow from analysis, interpretation, or data?																	
Type	Study	Y=Yes, N=No, U=Unclear, N/A=Not Applicable											Q_1_	Q_2_		Q_3_	Q_4_	Q_5_	Q_6_
Expert Opinion	Mehdi 2021 [61]												Y	Y		Y	Y	Y	U
	Yu 2018 [4]												Y	U		Y	Y	U	Y
		Q1: Source of opinion clearly identified? – Q2: Source of opinion has standing in the field of expertise? – Q3: Focus on the interests of the relevant population? – Q4: Opinion demonstrates a logically defended argument to support the conclusion drawn? – Q5: Reference to the extant literature? – Q6: Any incongruence with the literature/sources?																	

## Abbreviations

AVWP: Anterior Vaginal Wall Prolapse
BM: Bowel Movements
CD: Colonic Diverticulosis
COVID-19: Coronavirus Disease 2019
DPMDs: Defecation Postural Modification Devices
DTIs: Deep Tissue Injuries
FE: Finite Element
HADS: Hospital Anxiety and Depression Scale
JBI: Joanna Briggs Institute
LLL: Lower Leg Length
MeSH: Medical Subject Headings
OSI: Oscillation Stability Index
PICO: Patient/Population, Intervention, Comparison and Outcomes
PrI: Pressure Injury
PRISMA-ScR: Preferred Reporting Items for Systematic Reviews and Meta-Analyses extension for Scoping Reviews
PVR: Post-Void Residual
SBF: Span Between Feet
SBFmax: maximum Space Between Feet values
UTI: Urinary Tract Infection
VAS: Visual Analogue Scales
WHO: World Health Organization
